# Four Worlds

**Published:** 1888-04-07

**Authors:** 


### FOUR WORLDS.

I.

Oh! World of Beauty! it is good to live,
When all things smile on childhood's happy day;
Blue skies, warm sunshine, each their glory give
To gild our lives, ere clouds obscure our way.

II.

Oh! World of Freedom! it is good to strive,
With youth's bright energy of mind and heart,
To keep God's image in the world alive,
Against the tyrant take the weaker part.

III.

Oh! World of Labour! it is good to rest,
When mind and heart are hurt by daily strife;
To cast our cares and doubts on loving breast
Of Him who bore our cross in earthly life.

IV.

Oh! World of Sadness! it is good to die
When, after storms of sorrow, cometh peace;
Now, in our Father's keeping, restfully
Feeling that in His love our sorrows cease.

